# Adverse childhood events predicting cognitive complaint but not neuropsychological performance in older adults: Results from a large population-based study

**DOI:** 10.1192/j.eurpsy.2026.11567

**Published:** 2026-07-31

**Authors:** I. Rouch, C. Van de Leur, J.-M. Dorey, M.-P. Strippoli, S. Ranjbar, B. Laurent, M. Preisig, A. von Gunten

**Affiliations:** 1 Saint Etienne hospital, Saint Etienne; 2 U1219, INSERM, Bordeaux, France; 3 CEPP, CHUV, Lausanne, Switzerland; 4 CH le Vinatier, Bron; 5 U1028, INSERM, Lyon, France; 6 SUPPA, CHUV, Lausanne, Switzerland

## Abstract

**Introduction:**

Adverse childhood events (ACEs) have been associated with cognitive impairment in cross-sectional studies. However, the role of different ACEs on cognitive complaint and neuropsychological course in the general population remains under debate.

**Objectives:**

To prospectively assess the associations between specific types of ACEs with subjective cognitive complaint and the evolution of different neuropsychological scores in a large population-based cohort, adjusting for personality traits and depression and anxiety disorders

**Methods:**

Data stemmed from CoLaus|PsyCoLaus, a prospective population-based cohort study of initially 6734 participants followed-up for 20 years. The present sample included 1012 participants over 65 years. Diagnostic criteria for ACEs were elicited using semi-structured interviews; cognitive complaint was assessed with an auto-questionnaire. A neuropsychological battery was used to assessed memory, language, attention and executive functions. The relationship between the different ACEs and the risk of cognitive complaint was assessed with multiple logistic regression. Mixed Linear models measured the association between ACEs and the evolution of neuropsychological scores. The analyses were adjusted for age, sex, education, depression, anxiety disorders, and psychotropic drugs.

**Results:**

Participants with a history of childhood abuse or parental physical fighting exhibited an increased risk of having cognitive complaint in comparison to those without (respectively OR 4.35; p=0.002 and OR 1.73, p=0.03). On the other hand, our results did not show any relationship between ACEs and neuropsychological scores.

**Conclusions:**

Our results suggest that early trauma is associated with low self-esteem in cognitive skills, while normal neuropsychological performance. These findings highlight the importance of considering childhood adversity when examining cognitive complaint.

**Disclosure of Interest:**

None Declared

